# Investigation of the Possible Pharmacologically Active Forms of the Nicotinic Acetylcholine Receptor Agonist Anabaseine

**DOI:** 10.3390/md17110614

**Published:** 2019-10-29

**Authors:** Kristin Andrud, Hong Xing, Bjarne Gabrielsen, Linda Bloom, Vladimir Mahnir, Stephen Lee, Benedict T. Green, Jon Lindstrom, William Kem

**Affiliations:** 1 Department of Pharmacology and Therapeutics, College of Medicine, University of Florida, Gainesville, FL 32610, USA; Kristin.Andrud@du.edu (K.A.); hong.xing@ufl.edu (H.X.); wrkem@pharmacology.lufl.edu (V.M.); 2 Department of Chemistry, University of Florida, Gainesville, FL 32610, USA; bmlmpunta@yahoo.com; 3 Department of Biochemistry and Molecular Biology, College of Medicine, University of Florida, Gainesville, FL 32610; USA; lbloom@ufl.edu; 4 USDA-ARS Poisonous Plant Research Laboratory, Logan, UT 84341, USA; Stephen.Lee@ars.usda.gov (S.L.); Ben.Green@ars.usda.gov (B.T.G.); 5 Department of Neuroscience, University of Pennsylvania, Philadelphia, PA 19104, USA; jslkk@mail.med.upenn.edu

**Keywords:** acetylcholine, alkaloid, anabaseine, bipyridyl, cholinergic, nicotine, nicotinic acetylcholine receptor, ring-chain tautomerism, toxin

## Abstract

Three major forms of the nicotinic agonist toxin anabaseine (cyclic iminium, cyclic imine and the monocationic open-chain ammonium-ketone) co-exist in almost equal concentrations at physiological pH. We asked the question: Which of these forms is pharmacologically active? First, we investigated the pH dependence of anabaseine inhibition of [^3^H]-methylcarbamylcholine binding at rat brain α4β2 nicotinic acetylcholine receptors (nAChRs). These experiments indicated that one or both monocationic forms interact with the orthosteric binding site for ACh. However, since they occur at equal concentrations near physiological pH, we employed another approach, preparing a stable analog of each form and examining its agonist activities and binding affinities at several vertebrate brain and neuromuscular nAChRs. Only 2-(3-pyridyl)-1,4,5,6-tetrahydropyrimidine monohydrogen chloride (PTHP), the cyclic iminium analog, displayed nAChR potencies and binding affinities similar to anabaseine. The cyclic imine analog 2,3′-bipyridyl and the open-chain ammonium-ketone analog 5-methylamino-1-(3-pyridyl)-1-pentanone (MAPP), displayed ≤1% of the activity predicted if the one form was solely active. The lower potency of weakly basic 2,3′-bipyridyl can be explained by the presence of a small concentration of its monocationic form. Since the open chain ammonium-ketone monocationic form of anabaseine has some structural similarity to the neurotransmitter GABA, we also tested the ability of anabaseine and its 1,2-dehydropyrrolidinyl analog myosmine to activate a mammalian GABA_A_ receptor, but no activity was detected. We conclude that the monocationic cyclic iminium is the form which avidly binds and activates vertebrate nAChRs.

## 1. Introduction

Over eighty years ago, the Belgian pharmacologist Z. M. Bacq discovered the presence of a nicotine-like substance in a marine worm, the Atlantic hoplonemertine *Amphiporus lactifloreus* [1,2]. Small amounts of a weakly basic compound(s), called “amphiporine”, were partially purified using classical solvent extraction methods, but isolation by crystallization with standard alkaloid precipitating salts like picric acid was unsuccessful [3]. Anabaseine, an alkaloid with chemical and pharmacological properties similar to nicotine, was eventually isolated from the Pacific hoplonemertine *Paranemertes peregina* and detected in *A. lactifloreus* [4,5,6]. A piperideine analog of the tobacco alkaloid anabaseine, anabaseine also occurs in certain ants [7]. It is a potent agonist at a variety of nicotinic acetylcholine receptors, particularly the vertebrate skeletal muscle and α7 neuronal nAChRs that display high affinities for the snake toxin α-bungarotoxin. Anabaseine is also a weak partial agonist at α4β2 nAChRs that modulate cognitive and addiction neuronal circuits in the brain [8].

The imine bond of anabaseine, besides increasing its interaction with certain nAChRs, has enabled this molecule to serve as a useful “lead” compound for designing drug candidates that target α7 nAChRs [9,10,11]. 3-(2,4-dimethoxybenzylidene)anabaseine (also called DMXBA and GTS-21), the most studied anabaseine drug candidate, enhances cognition in humans [12,13], reduces inflammation [14], inhibits skeletal muscle wasting [15,16] and is neuroprotective in Parkinson’s [17] and Alzheimer’s disease [18] animal models. New anabaseine compounds that are more potent and selective α7 nAChR agonists have been reported [19,20]. 

Most nAChR agonists and competitive antagonists possess a cationic moiety that tightly binds within an electronegative “aromatic box” of the receptor formed by five aromatic amino acid side chains [21,22]. The dominant role of the monocationic form of nicotine has been demonstrated by experiments where the pH bathing intact skeletal muscle cells is varied [23,24,25]. Such an experimental approach assumes that altering pH mainly affects ionization of the ligand, rather than that of the nAChR. Subsequently several laboratories reported myriad effects of pH on several nAChR subtypes [26,27]. Altering extracellular pH was also found to affect muscle chloride permeability and resting membrane resistance, which indirectly affect response to nicotinic agonists [28,29].

**Figure 1:**
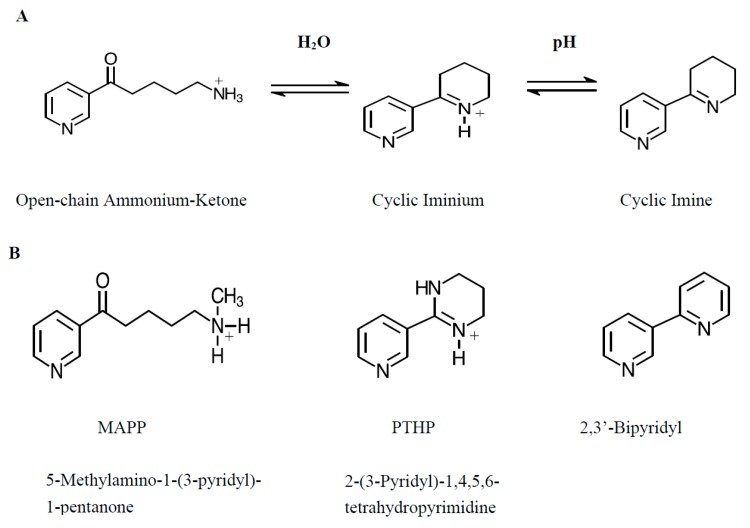
**A:** Structures of the three major forms of anabaseine that exist in dynamic equilibrium under physiological conditions; **B:** Anabaseine analogs synthesized to represent the particular form shown above. MAPP (also called *N*-methyl anabaseine) can still occur as a cyclic imine or iminium, but the equilibrium greatly (≥ 20-fold) favors the ammonium-ketone monocation form.

Whereas anabaseine contains a moderately basic (pKa 8.7, [30]) secondary amine group and mainly exists as a monocation at physiological pH, cyclic anabaseine contains a tertiary amine group but exists in three different chemical forms at neutral pH (Figure 1): the unionized cyclic imine (I), cationic cyclic iminium (I^+^) and monocationic open-chain ammonium ketone (AK^+^) forms are in dynamic equilibrium at approximately equal concentrations at pH 7.4 [31,32]. Our initial experiments on the pH dependence of anabaseine interaction with a brain nAChR, presented below, suggest that one or both monocationic forms are most active. However, since the two monocationic forms coexist at equal concentrations regardless of pH, a different experimental approach to assess their separate activities was required. Thus, we prepared a stable analog for each form and studied its effects on several vertebrate nAChRs: two fetal-type skeletal muscle (electric fish and human TE671 myosarcoma) receptors and the two major brain subtypes, α4β2 and α7. Since the open chain ammonium ketone cationic forms of anabaseine and its five-membered ring analog myosmine have some resemblance to another neurotransmitter, γ-aminobutyric acid (GABA), we also investigated whether anabaseine and its myosmine can activate a GABA_A_ receptor. 

## 2. Results

**Figure 2:**
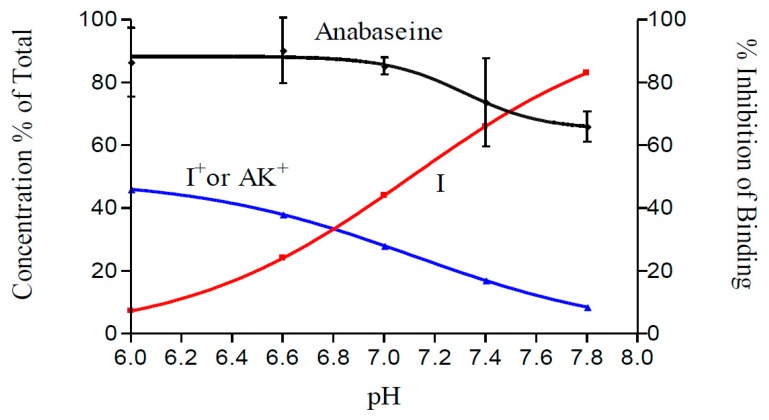
Concentrations of the three major forms of anabaseine as a function of pH as determined by UV spectrophotometry [32]. Left Ordinate: Concentration of each form expressed as % of the total anabaseine concentration. The red curve is the estimated cyclic imine concentration and the blue curve is the concentration of the cyclic iminium or the monocationic ammonium-ketone form of anabaseine (Assuming that K_H_ = 1.0, the concentrations of these ionized forms are equal). Right ordinate: pH dependence of anabaseine inhibition of [^3^H]-MCC binding to rat brain α4β2 receptors (Standard error bars included). Anabaseine inhibition (mean ± SEM) at each pH was the average of six replicate measurements.

Initially, we measured the pH-dependent binding of [^3^H]-methylcarbamylcholine ([^3^H]-MCC) to rat brain α4β2 nAChRs. In the absence of anabaseine we found a large (approximately 3-fold) enhancement of [^3^H]-MCC binding as pH increased from 6.0 to 7.8. While interesting in its own regard, since MCC is permanently ionized, this marked pH dependence of [^3^H]-MCC nAChR affinity complicated the analysis of the effect of pH on anabaseine binding, which was indirectly measured by [^3^H]-MCC displacement. Anabaseine inhibition of [^3^H]-MCC binding at each pH in Figure 2 is expressed with respect to the binding of [^3^H]-MCC alone at the same pH, so as to compensate for [^3^H]-MCC binding pH dependence. If only one or both monocationic forms of anabaseine are active, one would anticipate that inhibition of [^3^H]-MCC binding by the fixed concentration of anabaseine would be greatest at the low pHs and progressively decrease as the pH approaches and exceeds the pKa of its tertiary amine (iminium) group. However, if only the cyclic imine form binds with high affinity one would expect the opposite effect, an increase in the inhibition of [^3^H]-MCC binding as the pH approaches and exceeds the tertiary amine pKa. Anabaseine displacement of [^3^H]-MCC binding did decrease with increased pH, as expected for an active monocationic form, but the decrease was quantitatively less than would be predicted. Going from 5.8 to 7.8, the unionized cyclic imine (I) concentration was predicted [31] to increase approximately 20-fold, but the concentration of each monocationic form (I^+^ or AK^+^) would decrease ~4-fold. The pH dependence of anabaseine inhibition of MCC binding was not as marked as predicted for the monocationic form, but was certainly more consistent with the hypothesis that one or more monocationic forms of anabaseine is binding to this brain receptor. One additional complication in the interpretation of the results in Figure 2 is that specific receptor binding (and its inhibition) is not necessarily proportional to the displacing ligand (anabaseine) concentration, as it depends on a saturable binding isotherm (For instance, a Michaelis-Menton equation relating competing ligand concentrations and their respective equilibrium dissociation constants, which may not have the same pH dependence. The concentration of [^3^H]-MCC was 10 nM, which was almost the same as the K_d_ (11 nM) of this radioligand with rat brain membranes, under our experimental conditions [5]. 

Since the two monocationic forms show the same pH dependence and the same concentration when expressed as % of total anabaseine, another approach was required to assess their activities separately. We resorted to synthesizing and testing stable analogs that resemble the three anabaseine forms. PTHP, the tetrahydropyrimidinyl analog of the cyclic iminium form, is predicted to be >99% ionized at pH 7.4 and does not undergo ring opening within the physiological pH range considered here, since the basic (pK_a_ >10) amidinium group, unlike the imine group, is not susceptible to hydration and subsequent ring opening. 2,3′-bipyridyl (pK_a_ 4.4) was selected as an analog that possesses the basic structure of the unionized cyclic imine; at pH 7.4 >99.9% of the molecules of 2,3′-bipyridyl will be unionized. The monocationic ammonium-ketone open-chain form analog selected was 5-methylamino-1-(3-pyridyl)-1-pentanone, MAPP, shown in Figure 1; it mainly (>99%) exists in their open-chain form in water at physiological pH; its cyclic imine or iminium form, *N*-methylanabaseine, is present in very small concentration (≤5%) [31,32,33]. An unionized open-chain amino-ketone form which occurs at high pHs [32] can be neglected due to its very small predicted concentrations over the pH range 5.8 to 7.8 that is of interest here.

**Figure 3:**
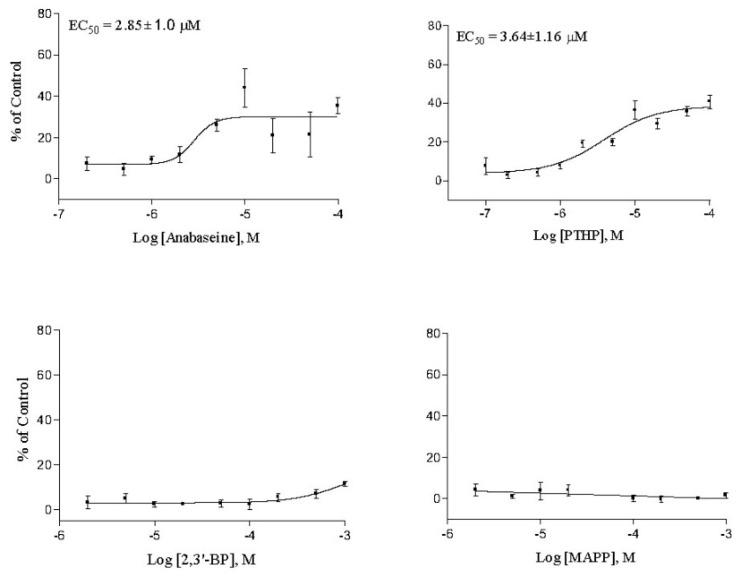
Activation of the human α4β2 neuronal nAChR expressed in tsA201 cells as measured by membrane depolarization using the FlexStation assay. Each point generally is the mean of four separate measurements. All responses were normalized with respect to the response of the cells to 0.5 μM epibatidine. Potency and efficacy estimates from these curves are found in Table 1.

**Figure 4:**
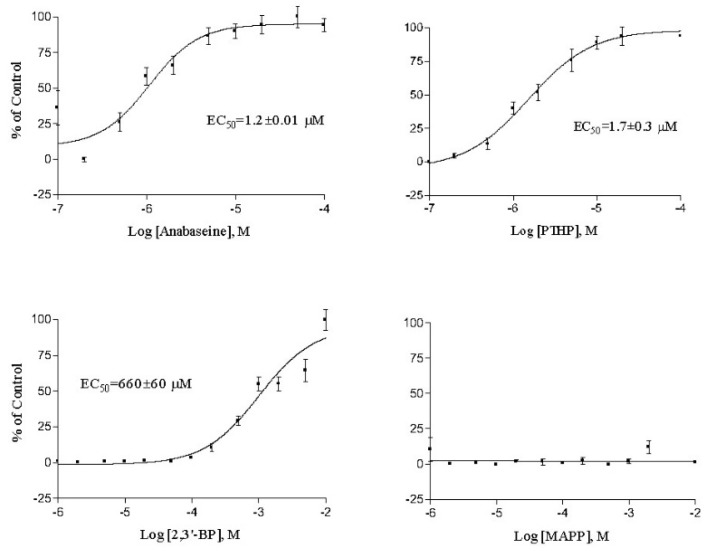
Activation of the human fetal (TE671 cells) skeletal muscle nAChR by the various stable anabaseine analogs measured by membrane depolarization using the FlexStation assay. Each point is generally the mean of four separate measurements. Responses were normalized to the 5 μM epibatidine response. Potency and efficacy estimates from these curves are found in Table 1.

PTHP was the only anabaseine analog that approached anabaseine in potency and binding affinity. Its potency and affinity for both the frog and human neuromuscular receptors was slightly inferior to that of anabaseine, but its agonist activity on vertebrate nAChRs had not been reported (Please see Figure 3 and Figure 4). The other analogs displayed less than 1% of anabaseine’s potency and binding affinity. The small potency of 2,3′-bipyridyl can be interpreted as being entirely due to its monocationic form, since the pKa of the most basic nitrogen on the 2′pyridyl ring is 4.4 [34].

**Figure 5:**
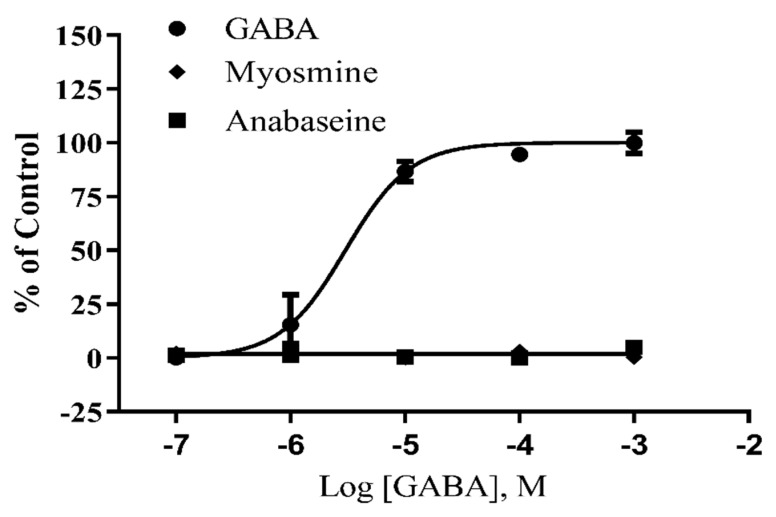
Investigation of possible activation of the human GABA_A_ receptor measured by membrane depolarization using a FlexStation assay. Each point is the mean of three separate measurements. All responses were normalized with respect to the response of the cells to 1 mM μM GABA. The EC_50_ value for GABA was 3.0 µM (95% confidence interval = 1.8–5.1 μM).

GABA_A_ receptors are homologous structures that have a common ancestry with nAChRs. Structure–activity studies on GABA_A_ receptors have shown that both the ammonium and carboxy groups are not absolutely required for agonist activity and the intermediate structure between these two ionizable groups can vary considerably as well—in some cases flexibility is minimized by a ring structure, such as in the agonist muscimol, and in other analogs the hydrocarbon chain is shortened or lengthened one methylene unit without loss of agonist activity. Since the open-chain cationic form of anabaseine resembles GABA by having a primary ammonium group that is separated a similar distance from a carbonyl group, we tested anabaseine and its pyrroline homolog myosmine on a human GABA_A_ receptor heterologously expressed in a HEK cell line [35]. Figure 5 shows that anabaseine and myosmine are not GABA_A_ agonists, at least on this particular GABA_A_ receptor subtype. 

## 3. Discussion

While most nAChR agonists and antagonists binding at ACh orthosteric sites are basic molecules with at least one ionizable N, there are exceptions, including lophotoxin [36], the neonicotinoids [37,38] and some recently synthesized pyrimidine agonists [39]. To optimize a molecule to serve as a drug or selective molecular probe of some receptor it is important to identify the form in which it interacts optimally with its target. In the case of anabaseine, our identification of the cyclic iminium form as the most active (and possibly the only) form suggests that structural modifications that enhance the basicity of its tetrahydropyridyl nitrogen will enhance potency and possibly selectivity for the intended receptor target. In fact, a major improvement in stability occurs with addition at the 3-position of the tetrahydropyridyl ring of an electron-conjugated system containing an aromatic ring, as in 3-(4-dimethylaminobenzylidene)-anabaseine [40] or 3-(2,4-dimethoxybenzylidene)-anabaseine, also called GTS-21 [41]. These anabaseine derivatives do not undergo ring opening (hydrolysis of the imine bond) under physiological conditions, due to their extended π electron conjugation [42]. 

Previously, it was assumed (but not experimentally tested) that the cyclic iminium form of anabaseine is the one which binds avidly to the ACh binding site in nAChRs [8]. This form was modeled to fit into a muscle type homology model [43]. The crystal structure of the molluscan acetylcholine binding protein binding anabaseine [44] actually was derived from crystals containing both the cyclic iminium and the open-chain monocationic form of anabaseine occupying some of the five identical binding sites. Unlike the cyclic iminium, the ammonium-ketone form ammonium group did not insert into the “aromatic box.” In the preparation of the AChBP-anabaseine crystals very high anabaseine concentrations were used so that most of the five sites would be occupied; therefore the presence of the ammonium-ketone form in the crystal structure does not necessarily indicate that this form of anabaseine would bind to AChBPs and nAChRs at the low concentrations of anabaseine that occur in our in vitro functional and binding studies.

The ability of a variety of 2-(aryl)-1,4,5,6-tetrahydropyrmidines to block neuromuscular nAChRs and produce teratogenic effects in chick embryos was reported long ago [45,46]. Some of these compounds were reported to be nAChR agonists. The only study of PTHP on neurons was that of Upshall et al. [47] on leech Retzius cells. Our results also indicate that PTHP is a potent agonist at neuronal as well as neuromuscular nAChRs. The 2-aryl compounds reported in the Brimblecombe et al. study did not include PTHP or other compounds having a 2-(3-pyridyl)- substituent, which is known to be essential for the agonist activity of nicotine and of anabaseine. For PTHP to be equipotent with anabaseine, based on the I^+^ form being the only active form, its EC_50_ would need to be ~1/3 of the EC_50_ of anabaseine, since the anabaseine (I^+^) is ~1/3 of its total concentration. The data for PTHP and anabaseine in Figure 3 and Figure 4 and in Table 1, indicate that PTHP has approximately 30% of the potency predicted for the anabaseine cyclic iminium form. One possible explanation for this slightly inferior potency is that the additional N on the tetrahydropyrmidinyl ring must form an H bond with some other atom within the aromatic box of the nAChR orthosteric binding site and this may be energetically unfavorable.

While 2,3′-bipyridyl displayed only a marginal potency at vertebrate receptors that could be predicted from its pK_a_, it has been found to be quite paralytic to insects [48] and crustaceans [49,50]. At least some arthropod nAChRs are known to not require a cationic ligand for activation, as shown by many studies with neonicotinoid insecticides such as imidachloprid [37]. These neonicotinoids also display a limited activity at vertebrate nAChRs [38]. It seems likely that 2,3′-bipyridyl affects some arthropod nAChRs through its unionized form. 

One can speculate as to what advantage(s) might accrue to an organism (in this case, a hoplonemertine or ant) to produce and secrete an imine-bond containing toxin like anabaseine. The entire body wall integument as well as the contiguous epithelium of the anterior proboscis of *P. peregrina* contains very high concentrations of anabaseine [5]. The proboscis everts and wraps around the prey during its capture allowing extensive contact of this epithelium with its annelid prey. The mineralized stylet of the proboscis can be observed to produce multiple punctures of the prey integument, which should further facilitate envenomation. In addition, potential predators will also be exposed in these ways to anabaseine and related alkaloids. Perhaps a major advantage of possessing an imine bond, besides an enhanced affinity for the nAChR orthosteric site [8], is its low basicity, which allows the unionized form to reach a relatively high concentration in the neutral pH range of the marine environment and thereby enhance passive diffusion across the integument of the prey or predator. The integument of a marine organism exposed to sea water is likely to have a pH near 8, well above the pH range in which anabaseine is primarily ionized. Thus, the toxin has a much better chance of entering the prey/predator than would anabaseine, the more basic secondary amine analog of anabaseine. 

Another consideration is how anabaseine is packaged within the venom gland cells [51]. It seems most likely that it is accumulated within secretory vesicles, which are generally acidic (pH 5–7). The ionized forms, especially the very polar open-chain forms, would be much less likely to diffuse out of the vesicles than would the cyclic imine form, so their dominance under these acidic conditions should enhance the energy efficiency of anabaseine storage. 

Thus, it is possible that each of the coexisting forms of anabaseine have functional significance, not only for understanding how this potent nAChR agonist interacts with its receptors, but they also may play different roles in storage and release of the toxin by the nemertine.

We have synthesized other anabaseine compounds whose properties will be reported in the future (Kem et al., in preparation). The current paper predicts that their relative pharmacological potencies will be affected by the extent that the cyclic iminium form dominates the equilibrium between these different forms. 

## 4. Materials and Methods

### 4.1. Compound Syntheses

2-(3-pyridyl)-1,4,5,6-tetrahydropyrimidine hydrochloride (PTHP): 3-pyridylamidine monohydrochloride (20 g, 0.127 mol) was dissolved in warmed absolute EtOH. To this solution was slowly added (dropwise over 5 min) freshly distilled 1,3-diaminopropane (9.42 g, 0.13 mol). This solution was refluxed for 6 h in a 500 mL round-bottomed flask. Afterwards the entire solution was placed on a steam bath to dryness. The crude solid was dissolved in approximately 500 mL hot isopropanol; crystals formed as the solvent was chilled to near 0 °C (6.2 g, 21.1% yield, MP 273–275°). Additional recrystallizations from the mother liquor raised the yield. Elemental Analysis: Found: % C = 54.71; % H = 6.17; % N = 21.22%. C_9_H_12_N_3_Cl requires: % C = 54.69; % H = 6.12; % N = 21.26. NMR Spectral data: In CD_3_OD, referenced to CD_3_OD at 3.34 ppm: δ (ppm): 8.85–9.19 (2H, m, H-2, 6; 8.23–8.55 (1H, dd, H-4); 7.85–7.93 (1H, m, H-5); 4.80 (2H, broad singlet, CD_3_OH); 3.64–3.87 (4H, t 4,6-CH_2_-); 1.97–2.43 (2H, quintet, 3-CH_2_-).

3-Pyridylamidine hydrochloride (PTHP Precursor): Our procedure was similar to that described by Schaefer and Peters [52] and Brown and Evans [53]. 3-cyanopyridine (35 g, 0.34 mol) was dissolved in 300 mL MeOH containing sodium methoxide (1.82 g, 0.034 mol). The flask was fitted with a condenser equipped with a drying tube and the clear solution stirred at 25 °C for 24 h. Ammonium chloride (19.8 g, 0.37 mol), added according to and the mixture was refluxed and stirred for 24 h. Not all (~30%) of the ammonium chloride dissolved. After cooling the solution to room temperature and removing excess NH_4_Cl by filtration the resulting solution was rotary evaporated and the product was dissolved in 100 mL hot isopropanol and filtered hot, leaving the NH_4_Cl behind. After 24 h at 0 °C the crystals were collected by filtration. Two more recrystallizations using isopropanol provided a total of 30 g product (MP 187–190 °C, 57% of theoretical yield). Elemental Analysis: Found, % C = 45.60; % H = 5.17/ % N = 26.61; C_6_H_8_N_3_Cl requires: % C = 45.73; % H = 5.12; % N = 26.66). NMR Spectral data: In CD_3_OD, referenced to CD_3_OD at 3.34 ppm: δ (ppm): 9.0 (2H, m, H-2, H-5,6 coupling of 5 H observed); 8.38–8.63 (1H, dd, H-4), H-4,5 coupling of 8 Hz observed, as is H-2,4 coupling of 1.5 Hz); 7.7–8.0 (1H, m, H-5); 5.3 (CD_3_OH, 4H, broad).

Anabaseine and 5-methylamino-1-(3-pyridyl)-1-pentanone (MAPP) dihydrochlorides (Figure 1) were synthesized as previously reported [31,32]. 2,3′-Bipyridyl was synthesized by silver acetate oxidation of anabaseine obtained from Sigma-Aldrich, St. Louis, MO, USA [49].

### 4.2. Radioligand Binding Assays

[^3^H]-carbamylcholine ([^3^H]-MCC) binding assays with rat brain membranes were carried out as previously reported with a final concentration of 0.5 nM [^3^H]-MCC [8,54]; 2.8 µM atropine sulfate (Sigma-Aldrich) was added to inhibit binding to muscarinic receptors. [^3^H]-Cytisine and [^125^I]-α-bungarotoxin ([^125^I]-α-BTX) were used in binding experiments with whole rat brain and TE671 cell membranes, respectively, according to Kem et al. [41]. Rat brain membranes (200 μg of protein) on TsA201 cell membranes (100 μg protein) expressing human α4β2 nAChRs were incubated with 0.5 nM [^3^H]-cytisine in a final volume of 500 µL of binding saline for 4 h at 5 °C. The experiments on α7 nAChRs rat brain membranes involved incubation with 0.5 nM [^125^I]-α-Btx for 3 h at 37 °C to assure that equilibrium was reached. Eight different concentrations of the experimental compound were usually tested in triplicate. Nonspecific binding was measured in the presence of 1 mM (S)-nicotine hydrogen tartrate (Sigma-Aldrich). Data were fitted using GraphPad Prism software (Version 4 GraphPad Software, San Diego, CA, USA) by nonlinear regression analyses to a sigmoidal one-site model with variable slope. Compound affinity for *Torpedo* electric organ membrane nAChRs was assessed by inhibition of 0.5 nM [^125^I]-α-BTX binding over a 30 min incubation period at 5 °C, since α-Btx binds irreversibly to skeletal muscle nAChRs. The IC_50_ was also estimated by the same Prism software.

### 4.3. Cell Culture

TsA201 cells expressing human α4β2 were maintained in media consisting of Dulbecco’s Modified Eagle medium supplemented with 10% FBS, 100 units/mL penicillin and 100 μg/mL streptomycin, 2 mM l-glutamine, 0.5 mg/mL zeocin and 0.6 mg/mL geneticin [55]. Cells were grown in 75 cm^2^ culture flasks, which were housed in a humidified incubator (Fisher Scientific, Atlanta, GA, USA at 37 °C in an atmosphere of 5% CO_2_. They were grown to around 80–90% confluence after harvesting with 0.25% trypsin and being split weekly at a subcultivation ratio of between 1:6 and 1:10.

### 4.4. Nicotinic Receptor FlexStation Functional Assays

Our experimental protocol was based on the initial study of Fitch et al. [56]. Cells were seeded at a density of roughly 5 × 10^4^ to 10^5^ cells/well in 96-well flat-bottom black wall culture plates coated with 50 μg /ml poly-d-lysine hydrobromide (Sigma-Aldrich, 70–150 kDa) and grown overnight in 100 μL culture medium. A proprietary membrane potential dye obtained from Molecular Devices (San Diego, CA, USA) was prepared by dissolving one bottle of dye into 30 mL of Hanks Saline (pH = 7.4) containing 20 mM HEPES buffer. The cells were incubated with 100 μL of dye for 30 min at 37 °C prior to the robotically controlled concentration-response experiment. Serial dilutions of a compound for dose-response analysis were prepared in 96-well plates by evaporation of a methanolic stock solution and then reconstituted in the appropriate volume of Hanks saline. Fluid transfer and readings were performed by a FlexStation fluorimeter (Molecular Devices). Excitation and emission wavelengths were set to 530 nm and 565 nm with a cutoff of 550 nm. The first 17 s were used as a basal reading. At 18 s, a test compound was added to determine the EC_50_, followed by addition of 25 μL KCl (40 mM final concentration) at 160 s to serve as a fluorescence calibrant. Compounds that had no measurable depolarizing activity on the TsA201 cells were then tested for their ability to inhibit a 5 µM control ACh response. Data were fitted and graphed with Prism (GraphPad) to determine the EC_50_.

### 4.5. GABA Receptor Flexstation Funcional Assays

WSS1 cells from ATCC (Manassas, VA, USA) which express functional GABA_A_ receptors were cultured as previously described by Wong et al. [57]. The cells were grown to near confluence and then transferred to black well, clear bottom 96-well assay plates (Corning Incorporated, Corning, NY, USA) 12–18 h prior to the assay. Molecular Devices blue dye (Sunnyvale, CA, USA) was reconstituted as previously described [58]. The WSS1 cells were allowed to come to room temperature, the culture medium was then aspirated, replaced with the blue dye and equilibrated for 30 min before being placed on a FlexStation 3 plate reader (Molecular Devices, Sunnyvale, CA, USA). Varying concentrations of γ-aminobutyric acid (GABA, Tocris Bioscience, Bristol, United Kingdom), myosmine (Sigma, St. Louis, MO, USA) and anabaseine [31,32] in blue dye solution were added to the cells by the FlexStation. Programmed readings and data analysis were performed as previously described [58,59]. The cellular responses to GABA were normalized to the maximum dye response generated by 1 mM GABA. The fifty percent effective concentration for GABA was determined using a sigmoidal dose-response equation (log(agonist) vs. normalized response—Variable slope) with Prism version 6.03 (GraphPad Software, Company, San Diego, CA, USA, www.graphpad.com).

## Figures and Tables

**Table 1:** Potencies and affinities characterizing the interactions of anabaseine and its stable analogs with several neuronal and neuromuscular nAChRs.

	Neuronal nAChR			Neuromuscular nAChR	
	Human	Rat	Rat	Human	*Torpedo*
Compound	α4β2	α4β	α7	TE671	Electric Organ
μM:	EC_50_	K_i_^1^	K_i_^2^	EC_50_	IC_50_^3^
Anabaseine	2.85 ± 1.0	0.096 ± 0.01	1.87 ± 0.10	1.2 ± 0.0	0.29 ± 0.18
PTHP	3.64 ± 1.2	0.38 ± 0.07	1.54 ± 0.76	1.7 ± 0.3	0.47 ± 0.01
2,3′-Bipyridyl	>1000	>50	>2300	660 ± 60	343 ± 48
MAPP	>2000	10	>2000	>1000	>500

^1^ Determined by displacement of [^3^H]-cytisine; ^2^ Determined by displacement of [^125^I]α-bungarotoxin; ^3^ Determined by inhibition of the rate of binding of [^125^I]-α-bungarotoxin.
